# Psychological and weight-related characteristics of patients with anorexia nervosa-restricting type who later develop bulimia nervosa

**DOI:** 10.1186/1751-0759-2-5

**Published:** 2008-02-12

**Authors:** Hiroki Nishimura, Gen Komaki, Tetsuya Ando, Toshihiro Nakahara, Takakazu Oka, Keisuke Kawai, Toshihiko Nagata, Aya Nishizono, Yuri Okamoto, Kenjiro Okabe, Masanori Koide, Chikara Yamaguchi, Satoshi Saito, Kazuyoshi Ohkuma, Katsutaro Nagata, Tetsuro Naruo, Masato Takii, Nobuo Kiriike, Toshio Ishikawa, Japanese Genetic Research Group for Eating Disorders

**Affiliations:** 1 Department of Psychosomatic Research, National Institute of Mental Health, National Center of Neurology and Psychiatry, 4-1-1 Ogawa-Higashi, Kodaira-shi, Tokyo 187-8553, Japan; 2 Department of Social Science and Medicine, Course for Health Science, Kagoshima University Graduate School of Medical and Dental Science, 8-35-1 Sakuragaoka, Kagoshima-shi, Kagoshima 890-8520, Japan; 3 Division of Psychosomatic Medicine, Department of Neurology, University of Occupational and Environmental Health, 1-1 Iseigaoka, Yahata-Nishi-ku, Kitakyushu-shi, Fukuoka 807-8555, Japan; 4 Department of Psychosomatic Medicine, Graduate School of Medical Sciences, Kyushu University, 3-1-1 Maidashi, Higashi-ku, Fukuoka-shi, Fukuoka 812-8582, Japan; 5 Department of Neuropsychiatry, Graduate School of Medicine, Osaka City University, 1-4-3 Asahimachi, Abeno-ku, Osaka-shi, Osaka 545-8585, Japan; 6 Tokyo Institute of Psychiatry, 2-1-8 Kamikitazawa, Setagaya-ku, Tokyo 156-8585, Japan; 7 Health Service Center, Hiroshima University, 1-7-1 Kagamiyama, Higashihiroshima-shi, Hiroshima 739-8521, Japan; 8 Department of Psychosomatic Medicine, Tenri Hospital, 200 Mishima-cho, Tenri-shi, Nara 632-8552, Japan; 9 Department of Internal Medicine, Hoshigaoka Maternity Hospital, 27 Inoue-cho, Chikusa-ku, Nagoya-shi, Aichi 464-0026, Japan; 10 Division of General Medicine and Neuropsychiatry, Aichi Medical University Hospital, Nagakute, Aichi 480-1195, Japan; 11 Department of Neuropsychiatry, School of Medicine, Sapporo Medical University, S-1, W-16, Chuo-ku, Sapporo-shi, Hokkaido 060-8543, Japan; 12 Department of Internal Medicine, Yufuin Kohseinenkin Hospital, 252 Kawaminami, Ufuin-cho, Ufu-shi, Oita, 879-5193, Japan; 13 Department of Psychosomatic Medicine, Hamamatsu University School of Medicine, 1-20-1 Handayam, Hamamatsu-shi, Shizuoka 431-3192, Japan; 14 Department of Psychosomatic Medicine, Nogami Hospital, 1-4-1 Komatsubara, Kagoshima-shi, Kagoshima 891-0114, Japan; 15 Department of Psychosomatic Medicine, Kohnodai Hospital, National Center of Neurology and Psychiatry, 1-7-1 Kohnodai, Ichikawa-shi, Chiba 272-8516, Japan

## Abstract

**Background:**

Patients with anorexia nervosa-restricting type (AN-R) sometimes develop accompanying bulimic symptoms or the full syndrome of bulimia nervosa (BN). If clinicians could predict who might change into the bulimic sub-type or BN, preventative steps could be taken. Therefore, we investigated anthropometric and psychological factors possibly associated with such changes.

**Method:**

All participants were from a study by the Japanese Genetic Research Group for Eating Disorders. Of 80 patients initially diagnosed with AN-R, 22 changed to the AN-Binge Eating/Purging Type (AN-BP) and 14 to BN for some period of time. The remaining 44 patients remained AN-R only from the onset to the investigation period. Variables compared by ANOVA included anthropometric measures, personality traits such as Multiple Perfectionism Scale scores and Temperament and Character Inventory scores, and Beck Depression Inventory-II scores.

**Results:**

In comparison with AN-R only patients, those who developed BN had significantly higher current BMI (p < 0.05) and maximum BMI in the past (p < 0.05). They also scored significantly higher for the psychological characteristic of parental criticism (p < 0.05) and lower in self-directedness (p < 0.05), which confirms previous reports, but these differences disappeared when the depression score was used as a co-variant. No significant differences were obtained for personality traits or depression among the AN-R only patients irrespective of their duration of illness.

**Conclusion:**

The present findings suggest a tendency toward obesity among patients who cross over from AN-R to BN. Low self-directedness and high parental criticism may be associated with the development of BN by patients with AN-R, although the differences may also be associated with depression.

## Background

Many eating disordered patients migrate between diagnostic categories, particularly from the initial diagnosis [1,2]. A diagnostic crossover from Anorexia Nervosa (AN) to Bulimia Nervosa (BN) is one of the most commonly observed [2,3]. Particularly for patients with initial diagnoses of AN restricting-type (AN-R), treatment is said to be difficult because many develop binging and vomiting. Previous studies of patients with an initial diagnosis of AN-R have reported that 62% changed to the AN binge-eating/purging type (AN-BP) [4] and 21~36% changed to BN [5,6]. These crossovers usually occurred within the first five years after onset of the eating disorder. Fichter et al. [7] found that patients crossed over from AN-R to AN-BP at a rate of 16.7% after two years, 10.3% after six years, and 3.7% after 12 years, and that patients crossed over from AN-R to BN at a rate of 13.3% after two years and 3.4% after six years.

These lines of evidence suggest that predicting the possibility of diagnostic crossover from AN-R at an early stage would be useful in treatment, because bulimia and purging behavior predict an unfavorable prognosis [8,9] and an increase in time to remission [5]. Only a few studies have examined the psychological factors possibly related to such crossovers from AN-R. Tozzi et al. [6] found that higher levels of 'Parental criticism' (a subscale of the Multidimensional Perfectionism Scale [MPS] for measuring perfectionism) and lower levels of 'Self-directedness' (a subscale of the Temperament and Character Inventory [TCI] for measuring personality) predicted crossover from AN-R to BN.

However, a relationship between these factors and depression has been documented [10,11]. Depression is generally prevalent among women with eating disorders [12,13]. Furthermore, several studies have suggested that patients with AN-BP are more depressive than those with AN-R [12,13], and that patients with BN are more depressive than those with AN [14]. Thus, it is possible that the degree of depression may be a major psychological factor related to the crossover from AN-R to BN. Indeed, the necessity of considering state variables such as depression in examinations of personality traits has been shown [10,15].

In addition, regarding the factors of body mass index (BMI) and obesity, patients with BN were higher than patients with AN-R on maximum BMI in the past [16,17] and childhood obesity is a reported risk factor for BN [1]. The relationship of a tendency to gain weight easily and the crossover from AN-R should be investigated.

Therefore, the aim of this study was to examine psychological factors and the tendency toward obesity related to the crossover from AN-R, and to examine the relationship between these psychological factors and depression.

## Method

### Participants

Patient data were collected from the institutions that participated in a study by the Japanese Genetic Research Group for Eating Disorders [18] (see Additional File 1). Participants were required to meet the following criteria: (1) agreement of participation/cooperation in the genetic analysis study after informed consent by the chief physician and agreement by the parents when a subject was under 20 years old, (2) diagnosis of an eating disorder based on the *Diagnostic and Statistical Manual of Mental Disorders, Fourth Edition *(DSM-IV), (3) duration of illness of the eating disorder of more than three months, (4) no current diagnosis of mental retardation (IQ < 70), organic brain disease, evident psychotic disorder (Schizophrenia, Bipolar Disorder) or somatic diseases that affect body weight, appetite, or eating. After applying these criteria, 373 patients were recruited [18]. From these patients, 25 for whom the details of the course of illness were unknown and 115 patients whose initial diagnosis was not AN-R were excluded. Of the remaining 233 patients, 153 were further excluded: (1) patients who crossed over to eating disorders not otherwise specified (n = 16), (2) patients who did not agree to fill out the questionnaires (n = 89) and those who did not complete all of the data-sets of the questionnaires (n = 38), (3) patients who had completely recovered at the time of the investigation (n = 10; 5 with AN-R only, 1 who crossed over from AN-R to AN-BP, and 4 who crossed over from AN-R to BN).

Thus, 80 patients who were all undergoing treatment for their illness at the time of the investigation (1 man and 79 women) were participants in the study. Their basic demographic variables (age at onset, age at investigation, duration of illness, current BMI, maximum BMI in the past, and minimum BMI in the past) were obtained for analysis. At the time of the investigation, their mean age was 24.00 ± 7.43 years old, duration of illness was 58.89 ± 53.14 months, and current BMI was 15.27 ± 3.05 kg/m^2^. There were no significant differences from those who were excluded from the current study (mean age, 22.13 ± 7.42 years old; duration of illness, 59.17 ± 64.24 months), except for current BMI (16.49 ± 3.63 kg/m^2^).

In accordance with the course of illness from the onset to the investigation period, the patients were classified into three groups: patients with AN-R only (n = 44), patients who crossed over from AN-R to AN-BP (n = 22), and patients who crossed over from AN-R to BN (n = 14). Patients who crossed over from AN-R to AN-BP included patients who returned to AN-R again after having developed AN-BP (n = 4; duration of AN-BP was 12–102 months). Patients who crossed over from AN-R to BN included patients who developed BN via AN-BP or who returned to AN-R again after having developed BN (n = 1; duration of BN was 7 months).

The current study was approved by the local ethics committees of the participating institutions and the National Center of Neurology and Psychiatry. Written informed consent was obtained after a full explanation of the study at each institution.

### Procedure

Informed consent was obtained from each participant. Clinical information such as 'age at onset', 'age at investigation', 'duration of illness', 'current BMI', 'maximum and minimum BMI in the past', and 'diagnostic crossover' was collected from the clinical records. The participants completed the following questionnaires at their treatment facility.

#### Multidimensional Perfectionism Scale (MPS)

The MPS [19] was used to evaluate multidimensional facets of perfectionism. The MPS is a 35-item questionnaire that measures six dimensions of perfectionism (Concern over mistakes, Personal standards, Parental expectations, Parental criticism, Doubt about actions, and Organization). Each item is scored on a five point scale ranging from 1 (strongly disagree) to 5 (strongly agree). A higher score indicates greater perfectionism. The MPS has demonstrated adequate reliability and high concurrent validity [19] and the Japanese version of MPS has also been validated [20].

#### Temperament and Character Inventory-125-4 (TCI)

The TCI is a 125-item inventory that consists of seven independent dimensions (Novelty seeking, Harm avoidance, Reward dependence, Persistence, Self-directedness, and Cooperativeness) that is based on a psychobiological model of personality [21]. Each item is scored on a four point scale ranging from 1 (disagree) to 4 (agree). The reliability and validity of the Japanese version of the TCI are well documented [22].

#### Beck Depression Inventory-II (BDI-II)

The BDI-II is a 21-item self-report questionnaire used to assess the severity of depression symptoms, based on the diagnostic criteria for depressive disorders in DSM-IV. Each item is scored from 0 to 3, with a higher score indicating greater intensity of the symptom. The total score is the sum of the items, ranging from 0 to 63; a higher score indicates greater depression. The reliability and validity of the Japanese version of the BDI-II are well documented [23].

### Statistical analyses

Data analysis was done with SPSS 11.0 J for Windows. Comparisons of more than three groups were performed with a one-way analysis of variance (ANOVA), and post hoc comparisons were performed with Dunnett's test or the Bonferroni test. To examine the effect of depression, one-way analyses of covariance (ANCOVA) were performed with the BDI-II scores entered as a covariate.

## Results

### Patient characteristics

Table 1 shows the clinical information, demographic data, and BDI-II scores of the three groups. The results of the ANOVA revealed significant differences among the three groups in duration of illness (p < 0.01), current BMI (p < 0.01), maximum BMI in the past (p < 0.05), and BDI-II score (p < 0.05). Post hoc comparisons using Dunnett's test with patients with AN-R only showed that the patients who crossed over from AN-R to AN-BP had significantly longer durations of illness than those with AN-R only (p < 0.05). Patients who crossed over from AN-R to BN had significantly higher current BMI (p < 0.05), maximum BMI in the past (p < 0.05), and BDI-II scores (p < 0.05) than those with AN-R only.

**Table 1:** Clinical information, demographic data, and BDI-II scores

	Patients with AN-R only			Patients who crossed over from AN-R to AN-BP			Patients who crossed over from AN-R to BN			
				
	Mean	(SD)	n	Mean	(SD)	n	Mean	(SD)	n	F values
Age at onset (yrs.)	18.66	(6.22)	42	19.60	(4.28)	22	17.55	(2.91)	13	0.62
Age (yrs.)	22.72	(8.10)	43	26.64	(7.05)	22	23.79	(4.66)	14	2.09
Duration of illness (mo)	42.55	(45.26)	44	84.45^a^	(63.33)	22	70.92	(40.70)	13	5.54**
Current BMI (kg/m^2^)	14.36	(2.14)	41	14.38	(1.32)	22	19.35^a^	(3.96)	14	24.96**
Maximum BMI (kg/m^2^)	19.97	(2.59)	42	20.83	(3.63)	20	22.73^a^	(2.92)	14	4.62*
Minimum BMI (kg/m^2^)	12.36	(1.96)	42	12.24	(1.68)	20	13.64	(1.93)	14	2.83
Differences of BMI(kg/m^2^)	7.61	(2.80)	42	8.60	(3.34)	20	9.09	(3.00)	14	1.61
BDI-II	18.75	(13.28)	44	22.36	(11.82)	22	29.36^a^	(12.22)	14	3.75*

Statistical significance by ANOVA: *p < .05, **p < .01

^a ^Superscript represents significant differences of p < .05 compared with patients with AN-R only by the Dunnett's test.

AN-R = Anorexia Nervosa Restricting-type, AN-BP = Anorexia Nervosa Binge-eating/purging type, BN = Bulimia Nervosa, BMI = Body Mass Index, BDI-II = Beck Depression Inventory-II, SD = standard deviation

### Comparisons of the MPS and TCI scores of the three groups

Table 2 shows the MPS and TCI scores of the three groups. The results of the ANOVA showed 'Parental criticism' on the MPS to be approaching significance (p < 0.10) and 'Self-directedness' on the TCI to be significant (p < 0.05). Post hoc comparisons using Dunnett's test with patients with AN-R only showed that patients who crossed over from AN-R to BN had significantly higher 'Parental criticism' scores (p < 0.05) and lower 'Self-directedness' scores (p < 0.05) than patients with AN-R only.

**Table 2:** Comparisons of the dimensions of the Multidimensional Perfectionism Scale and the Temperament and Character Inventory

	Patients with AN-R only		Patients who crossed over from AN-R to AN-BP		Patients who crossed over from AN-R to BN		
				
	n = 44		n = 22		n = 14		
				
	Mean	(SD)	Mean	(SD)	Mean	(SD)	F values
MPS							
Concern over mistakes	28.11	(6.99)	27.41	(10.27)	30.21	(7.06)	0.55
Personal standards	20.80	(5.18)	21.68	(6.25)	24.14	(5.95)	1.88
Parental expectations	11.11	(5.10)	12.55	(4.71)	14.14	(5.27)	2.08
Parental criticism	9.07	(3.86)	9.23	(3.12)	11.57^a^	(3.08)	2.77^†^
Doubts about actions	12.89	(3.64)	12.18	(3.38)	12.50	(2.85)	0.32
Organization	20.20	(3.86)	21.09	(4.16)	19.93	(3.25)	0.52
TCI							
Novelty seeking	45.55	(6.42)	45.55	(6.21)	49.00	(5.94)	1.75
Harm avoidance	60.70	(9.67)	60.73	(7.75)	64.79	(7.47)	1.23
Reward dependence	42.39	(5.33)	42.23	(4.99)	40.64	(5.67)	0.60
Persistence	13.61	(3.10)	15.23	(3.54)	14.29	(2.92)	1.88
Self-directedness	61.89	(11.17)	57.82	(10.33)	53.79^a^	(6.57)	3.62*
Cooperativeness	73.84	(8.03)	76.95	(6.71)	72.00	(9.96)	1.83
Self-transcendence	26.36	(7.31)	26.27	(8.31)	28.21	(5.03)	0.39

Statistical significance by ANOVA: ^†^p < .10, *p < .05

^a^Superscript represents significant differences of p < .05 compared with patients with AN-R only by the Dunnett's test.

AN-R = Anorexia Nervosa Restricting-type, AN-BP = Anorexia Nervosa Binge-eating/purging type, BN = Bulimia Nervosa, MPS = Multidimensional Perfectionism Scale, TCI = Temperament and Character Inventory, SD = standard deviation

These significant effects, however, disappeared in the ANCOVA in which BDI-II scores were entered as a covariate ('Parental criticism', F(2,80) = 1.69, p = 0.19; 'Self-directedness', F(2,80) = 0.95, p = 0.39).

### Examination of patients with AN-R only

To examine the effect of psychological factors related to the 'Duration of illness' of patients with AN-R only, we divided these patients into four categories based on the duration of illness: I, less than 1 year (n = 11); II, 1~2 years (n = 13); III, 3~5 years (n = 8); IV, more than 5 years (n = 12). Table 3 shows the BDI-II, MPS, and TCI scores of the four categories. The results of the ANOVA revealed a significant effect only for the MPS subscale 'Parental expectations' (p < 0.05). Post hoc comparisons using the Bonferroni test, however, showed that the four groups were not significantly different.

**Table 3:** Comparisons of the effect of duration of illness of patients with AN-R only on dimensions of the Multidimensional Perfectionism Scale, the Temperament and Character Inventory and the Beck Depression Inventory-II

Duration of illness	Less than 1 year		1–2 years		3–5 years		More than 5 years		
					
	n = 11		n = 13		n = 8		n = 12		
					
	Mean	(SD)	Mean	(SD)	Mean	(SD)	Mean	(SD)	F values
MPS									
Concern over mistakes	26.55	(4.18)	31.85	(7.39)	28.75	(8.43)	25.08	(6.40)	2.41
Personal standards	21.64	(6.47)	22.00	(3.79)	19.00	(6.50)	19.92	(4.34)	0.75
Parental expectations	8.55	(3.64)	13.15	(5.24)	8.25	(3.28)	13.17	(5.56)	3.69*
Parental criticism	8.18	(3.84)	9.62	(3.73)	8.38	(3.34)	9.75	(4.54)	0.47
Doubts about actions	14.27	(2.20)	12.69	(4.05)	12.88	(4.22)	11.83	(3.86)	0.87
Organization	20.73	(2.72)	20.62	(4.52)	19.75	(4.59)	19.58	(3.85)	0.24
TCI									
Novelty seeking	45.73	(7.27)	47.92	(5.79)	43.50	(7.65)	44.17	(5.22)	1.06
Harm avoidance	61.18	(10.23)	61.46	(8.52)	60.50	(8.80)	59.58	(11.83)	0.09
Reward dependence	41.09	(4.72)	41.00	(6.30)	46.00	(4.78)	42.67	(4.40)	1.85
Persistence	14.27	(3.13)	12.38	(2.40)	14.13	(4.64)	14.00	(2.45)	0.98
Self-directedness	61.45	(10.69)	56.54	(11.23)	65.88	(6.08)	65.42	(12.77)	1.84
Cooperativeness	74.91	(5.47)	69.31	(10.61)	77.25	(5.26)	75.50	(6.92)	2.28
Self-transcendence	31.09	(9.02)	24.15	(4.22)	25.13	(8.64)	25.25	(6.11)	2.30
BDI-II	19.27	(13.87)	21.46	(16.88)	18.25	(13.30)	15.67	(8.46)	0.39

Statistical significance: *p < .05

MPS = Multidimensional Perfectionism Scale, TCI = Temperament and Character Inventory, BDI-II = Beck Depression Inventory-II, SD = standard deviation

## Discussion

In comparison with patients with persistent AN-R, those who developed BN were significantly higher in both current BMI and maximum BMI in the past. They also scored significantly higher in 'Parental criticism' and lower in 'Self-directedness', but both these trait factors disappeared when depression scores were used as a co-variant. No significant differences were observed for any of the personality traits surveyed among patients with persistent AN-R irrespective of their duration of illness.

A tendency toward obesity among patients who cross over from AN-R to BN is reported by previous studies [16,17]. Because childhood obesity is a predisposition of BN [1], the present results suggest that patients with AN-R at onset who were overweight in the past are inclined to gain weight and will develop BN over the course of their illness. This finding is compatible with recent genetic studies [24]. The chromosomal region 10p, a susceptibility factor for BN, has been implicated in obesity [24] and the preproghrelin gene single nucleotide polymorphisms, another susceptibility factor for BN [18], were associated with higher current and maximum BMI in the past among female students [18,25]. In addition, the above genetic polymorphisms were related to higher 'Drive for Thinness-Body Dissatisfaction' scores measured by the Eating Disorder Inventory (EDI) [18,25].

Consistent with previous findings [6], high 'Parental criticism' and low 'Self-directedness' were associated with crossover from AN-R to BN in Japanese patients, indicating no ethnic or cultural effects on the psychological factors related to crossover from AN-R to BN. The finding of higher 'Parental criticism' scores of patients who crossed over from AN-R to BN is consistent with previous findings: lack of expressed empathy/affection from the parents predicted binge eating in AN-R patients [5]; stronger perception of deficits in parental nurturance in BN patients than in AN patients [26]; and 'Parental criticism' scores predicting bulimia scores measured by the EDI during a stressful situation for normal female high school students [27]. Families in which members are criticized may be weak in support for their members undergoing treatment, substantially inhibiting the treatment process and inclining restrictors to begin binging and vomiting. Moreover, maternal critical comments [28] and lack of parental care [29] are associated with poor outcomes in eating disorder patients. Thus, this study further suggests the importance of a supportive family system for the disease course of eating disordered patients.

The basic concept of 'Self-directedness' is "self-determination and 'willpower' or the ability of an individual to control, regulate, and adapt their behavior to fit the situation in accord with individually chosen goals and values"[21]. Patients with eating disorders who manifest low 'Self-directedness' are not capable of continuing the restriction behaviors that help them get thinner. Accordingly, these patients may begin binging and/or vomiting, which requires a change in diagnosis. Actually, 'Self-directedness' scores were lower in BN than AN-R [16,17,30]. Moreover, low 'Self-directedness' is associated with poorer outcome in AN patients [29,31]. Based on these lines of evidence, our findings suggest the importance of paying careful attention to 'Self-directedness' in the treatment process of AN-R patients.

We also observed the possibility that crossover from AN-R to BN is related to depression. While depression predicts future increases of binge eating in normal females [32-34] and in eating disorder patients [35], Tenconi et al. [36] reported that the degree of depression at baseline of patients with an initial diagnosis of AN-R did not predict the onset of binge eating. It is possible that depression may occur during the crossover period and that the depression may be caused by binging and vomiting. This hypothesis is supported by a study showing that the symptoms of eating-related concerns prospectively predict the onset of depression in adolescent girls [37]. In our study, relationships between depression and 'Parental criticism' and 'Self-directedness' were suggested. It is possible that trait factors such as 'Parental criticism' and 'Self-directedness' create a susceptibility to the development of depression, as low 'Self-directedness' was found to predict depression in normal undergraduates [38]. Otherwise, it is possible that the self-evaluation of trait factors was biased by a depressive mood [15,39]. The design of the present study, however, does not allow for an absolute determination of causation. Longitudinal investigation is necessary to confirm the relationship between crossover and the depression of patients with an initial diagnosis of AN-R.

In contrast to the findings for the patients who developed BN, patients who crossed over from AN-R to AN-BP showed no differences in psychological factors from those with AN-R only. This finding is supported by a cross-sectional study comparing the MPS scores of AN-BP and AN-R patients, where no significant differences were observed [40]. Regarding the TCI scores, however, the findings in different studies are not consistent: no significant differences between AN-R and AN-BP [13,16]; higher 'Novelty seeking' in AN-BP than AN-R [30,41,42]; and higher 'Self-directedness' in AN-R than AN-BP [41,42]. Because 'Novelty seeking' and 'Self-directedness' are reported to be associated with personality disorders [43,44] and alcohol abuse [45], it is possible that the co-morbidity of Axis I and II disorders merely contributes to those differences [30,41,42]. In addition, both of these AN sub-type groups were different in 'duration of illness'; this should be considered as a possible confounding factor, although there were not adequate numbers of subjects for a proper comparison. Further studies are needed regarding psychiatric co-morbidities and duration of illness among AN-R patients who develop AN-BP.

Scores on personality measures are also reported to be influenced by the degree of recovery from eating disorders [29,46]. In the present study, only those patients in an active state of illness were investigated. Although personality assessment and function might be influenced by the symptoms themselves (e.g., starvation) in an actively ill state [47], it is generally reported that personality is rather consistent [48,49], and temperament and character are independent of body weight in AN [41]. However, the number of patients excluded from our study because of their recovery at the time of the investigations was rather small; five with AN-R only, one who crossed over from AN-R to AN-BP, and four who crossed over from AN-R to BN. It is impossible to precisely compare the influence of symptoms of those in an active state of illness and those who have recovered.

Based on the recent findings that the highest rate of crossover was observed within the first five years after the onset of AN-R [5,6], we compared the patients who crossed over five years or more after the onset (n = 4) and those who crossed over within five years (n = 32). There were no significant associations of any of the psychological characteristics with the duration of illness and no significant differences on the MPS, TCI, or BDI-II between the two groups (data are not shown). Therefore, the influence of duration of AN-R is rather small on the psychological characteristics of these patients in the current study.

Finally, the present findings have clinical implications for the treatment of patients with eating disorders. Investigating the tendency toward obesity or a depressive state in a clinical setting will help us to predict crossover from AN-R to BN. Examining patient characteristics such as 'Parental criticism' and 'Self-directedness' may predict not only crossover but also, at least in part, the outcome of treatment. Cognitive and behavioral treatment, as well as nutritional interventions, for such risk factors will be necessary.

## Conclusion

The current results present the demographic and psychological characteristics of patients who cross over from AN-R to BN: a tendency toward obesity and the psychological characteristics of low self-directedness and high parental criticism. However, these psychological traits may also be associated with depression. Further research is needed to clarify the causal role of depression and its relationship to the identified psychological characteristics of patients who cross over.

## Competing interests

The author(s) declare that they have no competing interests.

## Authors' contributions

HN performed the statistical analysis and drafted the manuscript. GK conceived the study design, collected the clinical data set, and drafted the manuscript. TA participated in the design of the study and collected the clinical data set. TNakahara, TO, KK, T. Nagata, AN, YO, KOkabe, MK, CY, SS, KOhkuma, KN, TNaruo, MT, NK, TI and the Japanese Genetic Research Group for Eating Disorders collected the clinical data set. All authors read and approved the final manuscript.

## Supplementary Material

Additional file 1 Japanese Genetic Research Group for Eating Disorders. The investigators in this multi-site study group. Click here for file
